# The Relevance of Sleep Quality for Clinical Care in Parkinson’s Disease: Clinical and Cognitive Differences Between Good and Poor Sleepers: A Cross-Sectional Study

**DOI:** 10.3390/healthcare14162455

**Published:** 2026-08-09

**Authors:** Büşra Seçkinoğulları Korkusuz, Süleyman Korkusuz

**Affiliations:** 1 Department of Therapy and Rehabilitation, Kızılcahamam Vocational School of Health Services, Ankara University, Ankara 06890, Türkiye; 2 Department of Physical Therapy and Rehabilitation, Faculty of Health Sciences, Atılım University, Ankara 06830, Türkiye; suleyman.korkusuz@atilim.edu.tr

**Keywords:** Parkinson’s disease, sleep quality, cognition, executive function, visuospatial performance

## Abstract

**Highlights:**

**What are the main findings?**

- Parkinson’s disease patients with poor sleep quality showed greater disease severity and lower global cognitive, executive, and visuospatial performance than those with good sleep quality.
- Differences in cognitive performance were observed even in early-to-mid-stage Parkinson’s disease patients who were largely cognitively preserved.

**What are the implications of the main findings?**

- Subjective sleep quality assessment using the Pittsburgh Sleep Quality Index may help identify patients at higher risk of greater clinical and cognitive burden.
- Routine evaluation of sleep quality may support earlier recognition of vulnerable patients and contribute to more individualized, person-centered care in Parkinson’s disease.

**Abstract:**

**Background/Objectives**: This study aimed to compare disease severity and cognitive performance between Parkinson’s disease (PD) patients with good and poor sleep quality. **Methods**: This cross-sectional observational study included 69 patients with PD. Sleep quality was assessed using the Pittsburgh Sleep Quality Index (PSQI). Participants were classified as having good sleep quality (PSQI ≤ 5; *n* = 32) or poor sleep quality (PSQI > 5; *n* = 37). Disease severity was evaluated using the Unified Parkinson’s Disease Rating Scale (UPDRS) and Modified Hoehn and Yahr Scale (mH&Y). Global cognition, executive functions, and visuospatial performance were assessed using the Montreal Cognitive Assessment (MoCA), Stroop Test TBAG Form, and Clock Drawing Test, respectively. **Results**: Patients with poor sleep quality had significantly higher UPDRS scores (*p* = 0.004), longer disease duration (*p* = 0.043), and more advanced mH&Y (*p* < 0.001). They also demonstrated lower MoCA scores (*p* = 0.039) and lower Clock Drawing Test scores (*p* = 0.019). In addition, completion times on all Stroop test cards were significantly longer in the poor sleep quality group (*p* = 0.011–0.042), indicating poorer attention–executive performance. Moderate effect sizes were observed for disease severity and cognitive outcomes. **Conclusions**: Poor sleep quality was associated with greater disease severity and lower cognitive performance in early-to-mid-stage PD patients who were largely cognitively preserved. Routine assessment of subjective sleep quality may help identify patients with a higher clinical and cognitive burden and support more individualized, person-centered healthcare strategies in PD.

## 1. Introduction

Parkinson’s disease (PD) is a multi-systemic, progressive, and clinically heterogeneous neurodegenerative disease that goes beyond classic motor symptoms. While bradykinesia, rigidity, resting tremor, and postural instability are the most prominent motor features of the disease, non-motor symptoms often precede motor symptoms and constitute a significant portion of the disease burden, functional loss, and patient experience [1,2]. Cognitive impairments, mood symptoms, autonomic dysfunction, pain, and sleep disturbances are among the major components of this broad spectrum of non-motor symptoms. However, these symptoms tend to go unnoticed unless systematically assessed in clinical management [2,3].

Sleep disturbances are among the frequently observed non-motor symptoms in PD, both during the prodromal and established phases. The literature reports a prevalence of poor sleep quality of 58.07%, and it is associated with more severe depressive and anxious symptoms, lower cognitive performance, and more pronounced motor findings [4,5]. A recent comprehensive review highlights that insomnia, excessive daytime sleepiness, circadian rhythm disorders, obstructive sleep apnea, restless legs syndrome, and REM sleep behavior disorder are common in Parkinson’s patients; that these problems worsen as the disease progresses; and that they negatively affect quality of life. Furthermore, it is reported that sleep problems can develop in approximately 80% of patients within about five years of diagnosis [4]. Studies in early-stage PD patients show that sleep disturbances are present from the onset of the disease and often exhibit a complex pattern where multiple sleep problems occur together [6].

In addition to sleep disturbances, cognitive impairment is another common non-motor feature in PD that significantly contributes to the clinical burden. Current data indicate that mild cognitive impairment accompanying PD can be seen in approximately 30% of early-stage cases and can reach up to 75% in follow-ups longer than 10 years. Executive functions, attention, visuospatial skills, and memory are particularly affected [7]. In clinical practice, although this condition may appear independent of motor symptoms, it directly affects daily living activities, self-care, adherence to treatment, and coping mechanisms. Therefore, cognitive assessment in PD is necessary not only for dementia screening but also for detecting early and milder functional impairments [8,9].

The relationship between sleep quality and cognitive function is receiving increasing attention in PD. Poor sleep quality is reported to be associated with lower performance in global cognition, memory, and executive functions [10]. In addition, sleep problems such as REM sleep behavior disorder, excessive daytime sleepiness, and insomnia are associated with cognitive impairment [11,12].

The relationship between sleep quality and disease severity is also clinically important. The literature reports that poor sleep quality is associated with greater impairment in daily living activities, that disease severity may play a mediating role in this relationship, and that cognitive status may have a protective effect [13]. These findings suggest that sleep, cognition, and disease severity should be considered not as independent but as interacting clinical components. Considering the growing emphasis on person-centered care in PD, identifying sleep-related clinical and cognitive differences may help clinicians better tailor monitoring and management strategies to individual patient needs.

The current literature suggests that sleep disturbances in PD may be linked to cognitive functions, daily living performance, and various disease-related clinical outcomes. However, most previous studies have focused on specific sleep disturbances or assessed only certain areas of cognitive function. Furthermore, studies that comprehensively assess global cognitive status, executive functions, visuospatial performance, and disease severity in patients with PD classified according to sleep quality are limited. Therefore, the present study aims to identify differences in disease severity, global cognitive status, executive functions, and visuospatial performance according to sleep quality in patients with PD. We hypothesized that patients with poor sleep quality would exhibit greater disease severity than those with good sleep quality. We further hypothesized that poor sleep quality would be associated with lower global cognitive performance, executive function, and visuospatial performance.

## 2. Materials and Methods

### 2.1. Research Design and Participants

This study was designed as an observational and cross-sectional study to examine differences in disease severity and cognitive performance based on sleep quality in patients with PD. Sixty-nine patients with PD who presented to the neurology outpatient clinic and met the inclusion criteria were included in the study. The reporting of the study was based on the recommendations of the Strengthening the Reporting of Observational Studies in Epidemiology (STROBE). Ethical approval was obtained from the Atılım University Human Research Ethics Committee (Decision No: E-59394181-604.01-113629). The study was registered in the ClinicalTrials.gov database under the number NCT07536503. Institutional permission for participant recruitment at the Neurology Outpatient Clinic of Hacettepe University Hospital was obtained before study initiation. All participants provided written informed consent before the study.

To reduce potential selection bias, inclusion and exclusion criteria were defined in advance and applied to all participants in a standard manner. Participants were selected using sequential sampling from among patients with PD followed up at the Neurology Outpatient Clinic of Hacettepe University Hospital. Recruitment was performed between May and June 2026. Inclusion criteria were being between 40 and 70 years of age, having been diagnosed with PD by a neurologist according to the UK Parkinson’s Disease Brain Bank diagnostic criteria, having a mH&Y stage between 1 and 3, and having a MoCA-Turkish score of ≥21. Individuals with severe visual or hearing impairment, communication problems severe enough to prevent completion of the assessments, a history of concomitant neurological disease, congestive heart failure, and serious systemic diseases that could affect assessment results were excluded from the study.

### 2.2. Procedures

Eligible participants were informed about the study and then evaluated. All evaluations were conducted during the “on” period, approximately 1 h after participants took their antiparkinsonian medication, to minimize the effect of motor fluctuations. The evaluations were completed in a single session on the same day. Participants’ age, gender, education level, and disease duration were recorded using a demographic data form. Subsequently, sleep quality, disease severity, disease stage, and cognitive assessments were performed.

Sleep quality was determined as the primary outcome variable, while disease severity, disease stage, global cognitive status, attention–executive functions, and visuospatial performance were defined as secondary outcome variables.

#### 2.2.1. Staging of Parkinson’s Disease and Disease Severity

Disease severity was assessed using the Unified Parkinson’s Disease Rating Scale (UPDRS) [14]. The UPDRS consists of four parts: mentation, behavior and mood (Part I); activities of daily living (Part II); motor examination (Part III); and complications of therapy (Part IV). Higher scores indicate greater disease severity. The total UPDRS score was used in the analyses.

Disease stage was determined using the mH&Y [15]. The mH&Y scale is a widely used clinical staging system for assessing the severity of motor symptoms and the level of disease progression in PD. Higher stages on the scale indicate more advanced disease severity.

#### 2.2.2. Pittsburgh Sleep Quality Index

Participants’ subjective sleep quality was assessed using the Pittsburgh Sleep Quality Index (PSQI) [16]. The Turkish validity and reliability study of the scale was conducted by Ağargün et al. [17]. The PSQI consists of 19 self-report items evaluating sleep characteristics over the past month. The scale comprises seven components: subjective sleep quality, sleep latency, sleep duration, habitual sleep efficiency, sleep disorders, sleep medication use, and daytime dysfunction. Each component is scored between 0 and 3, and the total score ranges from 0 to 21. Higher scores indicate worse sleep quality. Participants were divided into two groups based on their PSQI global score. Individuals with a PSQI score of ≤5 were classified as having good sleep quality, whereas those with a PSQI score of >5 were classified as having poor sleep quality.

#### 2.2.3. Montreal Cognitive Assessment Scale

Global cognitive status was assessed using the MoCA [9]. The Turkish validity and reliability study of the scale in patients with PD was conducted by Ozdilek and Kenangil [18]. The MoCA is a 30-point assessment test that evaluates attention, executive functions, memory, language, abstraction, visual–spatial skills, and orientation. Higher scores indicate better cognitive performance.

#### 2.2.4. Stroop Test TBAG Form

Attention and executive functions were assessed using the Stroop Test TBAG Form [19]. The test evaluates executive function processes such as selective attention, cognitive flexibility, response inhibition, and interference control. Adaptation to Turkish culture, validity, and reliability studies have been conducted on the test, showing that it can be used particularly in evaluating attention processes and resistance to disruptive effects. In this study, the completion time for each card of the Stroop Test TBAG Form was recorded. Longer completion times were interpreted as lower attention and executive function performance.

#### 2.2.5. Clock Drawing Test

Visual–spatial skills and executive functions were assessed using the Clock Drawing Test [20]. Participants were asked to place clock numbers inside an empty circle and indicate the specified time. The test evaluates visual–spatial organization, planning, and executive functions. Scoring was performed according to the method recommended for the Turkish version [21]. Higher scores indicate better performance.

### 2.3. Statistical Analysis

Data were analyzed using IBM SPSS Statistics for Windows, Version 23 (IBM Corp., Armonk, NY, USA). The normality of continuous variables was assessed using the Shapiro–Wilk test together with skewness and kurtosis statistics, histograms, and Q–Q plots. Continuous variables were presented as mean ± standard deviation (SD) or median (minimum–maximum), whereas categorical variables were expressed as frequencies and percentages. Participants were classified into good and poor sleep quality groups according to the PSQI cutoff score of 5. Continuous variables showing an approximately normal distribution in both groups were compared using the independent-samples *t*-test. In contrast, variables that violated the normality assumption were compared using the Mann–Whitney U test. Categorical variables were analyzed using the chi-square test or Fisher’s exact test, as appropriate. Statistical significance was set at *p* < 0.05. To determine whether differences in cognitive outcomes remained after adjustment for potential confounding factors, analyses of covariance (ANCOVA) were performed with sleep-quality group as the fixed factor and age, disease duration, and Modified Hoehn and Yahr stage as covariates. Prior to ANCOVA, the assumptions of linearity, homogeneity of regression slopes, homogeneity of variances, and normality of residuals were evaluated and found to be satisfied. No missing data were observed; therefore, all enrolled participants were included in the analyses. Because the analyses evaluated distinct, prespecified outcome variables representing different clinical domains (disease severity, global cognition, executive function, and visuospatial performance), rather than multiple tests of a single outcome, no formal adjustment for multiple comparisons was applied.

## 3. Results

A total of 69 patients with PD were included in the study. According to the Pittsburgh Sleep Quality Index (PSQI), 32 patients were classified as having good sleep quality and 37 patients as having poor sleep quality. The demographic characteristics of the participants are presented in Table 1. There were no significant differences between the groups regarding age (*p* = 0.217), body mass index (BMI) (*p* = 0.462), sex distribution (*p* = 0.788), or educational level (*p* = 0.348) (Table 1).

**Figure 1:**
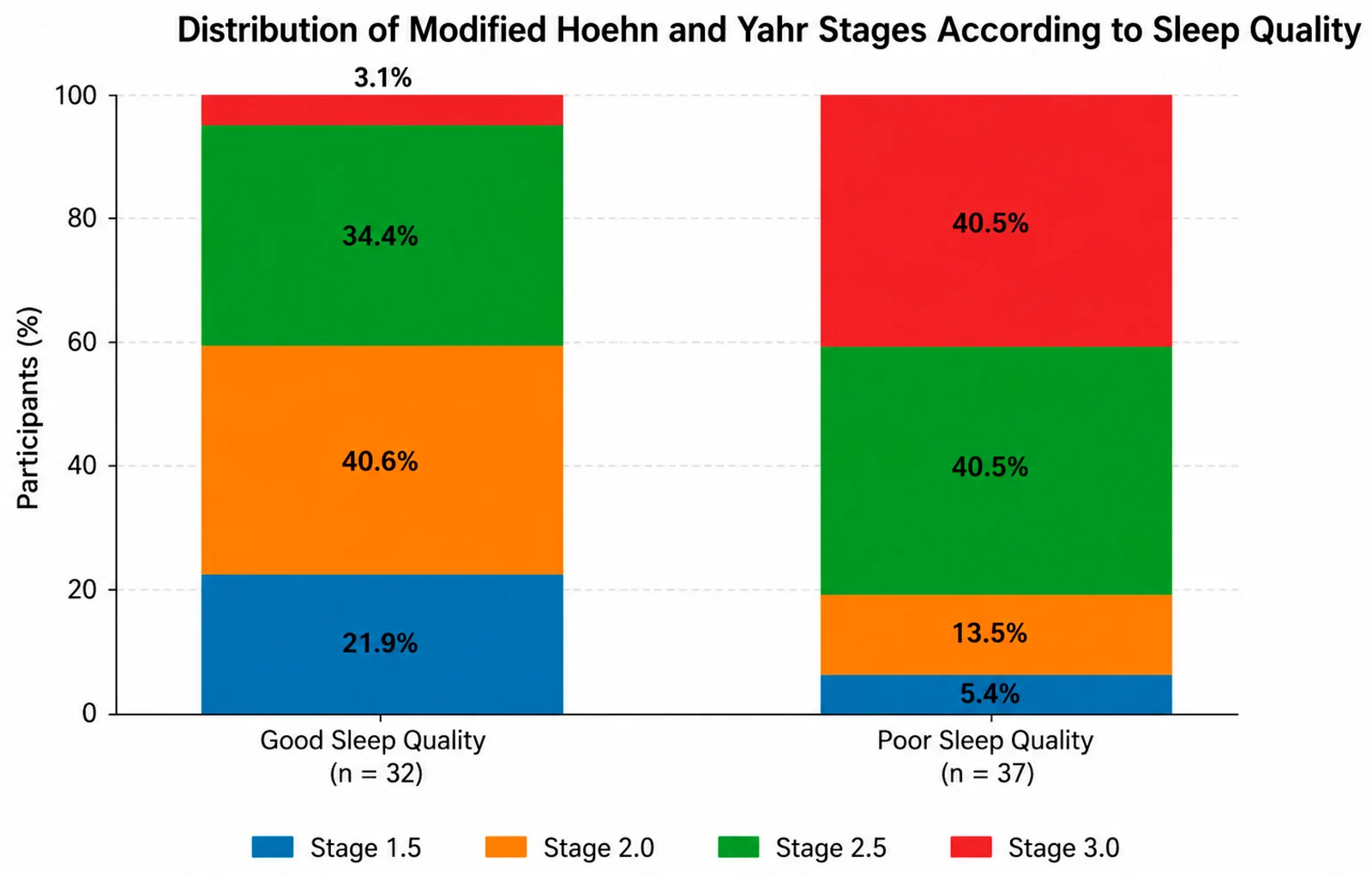
Distribution of Modified Hoehn and Yahr Scale stages according to sleep quality.

Clinical characteristics according to sleep quality are presented in Table 2. No significant differences were found between the groups regarding dominant extremity (*p* = 0.657) or disease onset side (*p* = 0.074). However, patients with poor sleep quality had significantly higher UPDRS scores (37.19 ± 9.18 vs. 31.16 ± 7.44, *p* = 0.004), longer disease duration (7.29 ± 4.81 vs. 5.28 ± 3.24 years, *p* = 0.043), and more advanced mH&Y stages (*p* < 0.001) (Figure 1) than those with good sleep quality (Table 2).

Regarding cognitive performance, patients with poor sleep quality demonstrated significantly lower MoCA scores than patients with good sleep quality (24.40 ± 1.62 vs. 25.28 ± 1.83, *p* = 0.039). Similarly, Clock Drawing Test scores were significantly lower in the poor sleep quality group (5.54 ± 1.78 vs. 6.50 ± 1.52, *p* = 0.019). (Table 3).

For executive functions, patients with poor sleep quality required significantly longer completion times on all Stroop test cards. Significant differences were observed for Stroop Card 1 (*p* = 0.039), Stroop Card 2 (*p* = 0.042), Stroop Card 3 (*p* = 0.027), Stroop Card 4 (*p* = 0.032), and Stroop Card 5 (*p* = 0.011) (Table 3).

To determine whether the observed differences in cognitive performance remained after adjustment for age, disease duration, and Modified Hoehn and Yahr stage, analyses of covariance (ANCOVA) were performed (Table 4). After adjustment, patients with poor sleep quality continued to demonstrate significantly lower cognitive performance than those with good sleep quality. Specifically, the poor sleep quality group had significantly lower adjusted MoCA scores (adjusted mean: 24.304 vs. 25.399; *F* = 5.126, *p* = 0.027, $\eta_{p}^{2}$ = 0.074). They also required significantly longer completion times on the Stroop Test, including Stroop-1 (*F* = 4.536, *p* = 0.037, $\eta_{p}^{2}$ = 0.066), Stroop-2 (*F* = 7.944, *p* = 0.006, $\eta_{p}^{2}$ = 0.110), Stroop-3 (*F* = 6.257, *p* = 0.015, $\eta_{p}^{2}$ = 0.089), Stroop-4 (*F* = 7.101, *p* = 0.010, $\eta_{p}^{2}$ = 0.100), and Stroop-5 (*F* = 7.399, *p* = 0.008, $\eta_{p}^{2}$ = 0.104). In addition, the poor sleep quality group had significantly lower adjusted Clock Drawing Test scores than the good sleep quality group (*F* = 5.875, *p* = 0.018, $\eta_{p}^{2}$ = 0.084).

Across the ANCOVA models, age, disease duration, and modified Hoehn and Yahr stage were generally not significant independent predictors of cognitive performance, with the exception of age in the Stroop-3 model. Multicollinearity diagnostics showed low variance inflation factors (VIFs = 1.02–1.42) and high tolerance values (0.71–0.98), indicating no evidence of problematic multicollinearity among the covariates (Supplementary Tables S1 and S2). Accordingly, the observed adjusted group differences are unlikely to be explained by problematic multicollinearity among the included covariates.

## 4. Discussion

In this study, individuals with PD were divided into two groups based on their PSQI scores: those with good sleep quality and those with poor sleep quality. These groups were then compared in terms of disease severity, global cognitive status, executive functions, and visuospatial performance. The main finding of the study was that individuals with poor sleep quality showed higher disease severity and lower cognitive performance compared to those with good sleep quality. Specifically, global cognition, executive functions, and visuospatial performance were significantly lower in individuals with poor sleep quality. Importantly, these differences remained statistically significant after adjustment for age, disease duration, and mH&Y. This indicates that the observed differences in cognitive performance were not fully explained by these clinical characteristics and remained statistically significant after adjustment for age, disease duration, and Modified Hoehn and Yahr stage. These results suggest that sleep quality is not only a common non-motor symptom in PD but may also be closely related to the clinical and cognitive burden of the disease.

Our cognitive findings are consistent with previous studies. In a systematic review by Marafioti et al. [22], sleep disorders were reported to be associated with impairment in executive functions, attention processes, visual-constructive skills, and episodic memory. Similarly, Nagy et al. [23] showed that cognitive impairment in Parkinson’s patients with REM sleep behavior disorder initially manifested in the areas of attention and memory, and over time expanded to include executive functions and visual–spatial skills. Furthermore, meta-analytic data reveal that poor sleep quality is associated with lower performance in global cognition, verbal memory, and executive functions [10]. More recent studies, in particular, show that REM sleep behavior disorder and excessive daytime sleepiness can accelerate cognitive decline [11,12].

There are several biological mechanisms that may explain the differences observed in cognitive function with poor sleep quality in PD. Sleep plays a critical role in synaptic plasticity, memory consolidation, and the reorganization of neural networks [24,25]. Therefore, chronic sleep disturbance can exacerbate neurodegenerative processes and negatively affect performance in areas such as executive functions, visuospatial skills, and global cognition [26]. Given that PD affects the frontostriatal and extrapyramidal networks involved in cognitive function, sleep disturbances may increase existing neural fragility, making cognitive impairment more pronounced [27,28]. These mechanisms may partially explain the lower performance observed in different cognitive domains in individuals with poor sleep quality in our study.

The observed differences in global cognition, executive function, and visuospatial performance remained statistically significant after adjustment for age, disease duration, and mH&Y. Although disease progression and advancing age are well-recognized determinants of cognitive performance in PD, these adjusted analyses indicate that the observed group differences were not fully explained by these clinical characteristics. Therefore, the present findings suggest that poorer subjective sleep quality may reflect differences in cognitive performance beyond those attributable to age, disease duration, and disease stage alone. Nevertheless, because of the cross-sectional design, these adjusted results should be interpreted as demonstrating persistent group differences rather than causal or independent effects. Among the covariates, only age was significantly associated with Stroop-3 performance, whereas disease duration and Modified Hoehn and Yahr stage were not significantly associated with the adjusted cognitive outcomes. Furthermore, multicollinearity diagnostics indicated no evidence of problematic multicollinearity among the covariates, suggesting that the adjusted parameter estimates were stable.

Our study’s finding that individuals with poor sleep quality had lower executive function performance supports the findings reported in the literature. Papp et al. [29] reported that changes in REM sleep duration were associated with verbal fluency performance. Since verbal fluency is considered a significant component of executive functions, this finding supports the link between sleep characteristics and executive function performance. Similarly, Kong et al.’s [26] systematic review and meta-analysis showed that various sleep problems, such as poor sleep quality, sleep fragmentation, decreased sleep efficiency, and circadian rhythm disturbances, were associated with impairment, particularly in executive function areas such as attention, working memory, cognitive flexibility, and problem-solving. Therefore, our results suggest that impaired sleep quality may negatively affect cognitive processes associated with frontal-subcortical networks in patients with PD.

Another important finding is that individuals with poor sleep quality have lower visual–spatial performance. While Marafioti et al. [22] reported that sleep disorders are associated with impairment in visual-constructive skills, Nagy et al. [23] showed that visual–spatial areas may be more significantly affected in individuals with REM sleep behavior disorder in later stages. The results obtained in the present study support the idea that impaired sleep quality in PD can affect not only a single cognitive area but also different cognitive areas.

It is noteworthy that our study found higher disease severity in individuals with poor sleep quality. In PD, numerous sleep problems such as insomnia, REM sleep behavior disorder, excessive daytime sleepiness, restless legs syndrome, and sleep-related breathing disorders are known to be common [30,31]. It has been reported that these disorders can be seen even in the prodromal and early stages and may become more pronounced as the disease progresses [32,33,34,35]. Dodet et al. [6] showed that approximately half of early-stage patients with PD have multiple sleep disorders, and the number of sleep disorders increases with disease duration. Furthermore, it has been reported that the prevalence of poor sleep quality is approximately 58% and is associated with more severe motor symptoms, lower cognitive performance, and more pronounced psychological impact [5]. Similarly, Luo et al. [13] showed that poor sleep quality negatively affects daily living activities, that disease severity plays a mediating role in this effect, and that better cognitive status can partially mitigate this negative impact. When this information is considered together with our study results, it is thought that sleep quality should be evaluated not only as a symptom in PD but also as an important characteristic that can affect the clinical presentation of the disease and the functional status of the individual. Although disease severity differed between the groups, the persistence of cognitive differences after adjustment for age, disease duration, and mH&Y stage suggests that these findings cannot be explained solely by differences in disease progression.

One of the remarkable aspects of the current study is that these findings were obtained in early-to-mid-stage PD patients and in individuals who were largely cognitively preserved. Although cognitive impairment is expected to occur more in the later stages of the disease, our results showed that individuals with poor sleep quality scored lower in global cognition, executive functions, and visuospatial performance. This suggests that cognitive differences based on sleep quality may also be observed in the early stages of the disease. A significant aspect of this study is that it classifies patients with PD into groups with good and poor sleep quality based on their subjective sleep quality and evaluates disease severity, global cognition, executive functions, and visuospatial performance together. While most studies in the literature focus on specific sleep disorders such as REM sleep behavior disorder, excessive daytime sleepiness, or polysomnographic changes, studies that evaluate different cognitive domains and disease severity together in patients with PD classified according to sleep quality are limited. Therefore, the current study contributes to the literature by being based on a subjective sleep quality approach that can be easily used in clinical practice. Furthermore, the ease of application and low cost of the PSQI increases the transferability of the findings to clinical practice. This study has several strengths. First, sleep quality was assessed using a scale with proven validity and reliability. Second, cognitive performance was assessed not only at the global cognition level but also for different cognitive domains such as executive functions and visuospatial skills, which are known to be susceptible to impact in PD. Thus, it did not rely solely on a global cognitive screening result but presented a more comprehensive cognitive profile based on sleep quality. Third, the combined assessment of disease severity and cognitive performance allowed for a more comprehensive demonstration of the clinical significance of sleep quality. Finally, in our study, differences in disease severity and cognitive performance were demonstrated using the PSQI, an easily applicable and low-cost clinical tool that assesses subjective sleep quality. This finding suggests that subjective sleep assessments may be useful in identifying individuals who may exhibit lower cognitive performance in clinical practice without resorting to more complex and costly assessments such as polysomnography.

However, this study has several limitations. First, because of the cross-sectional design and the potential confounding effect of disease severity, the present findings should be interpreted as demonstrating an association rather than providing evidence that poor sleep causes cognitive impairment. Second, sleep quality was assessed solely using the self-reported PSQI. Objective sleep assessment methods, such as polysomnography or actigraphy, which provide detailed information on sleep architecture and sleep-stage distribution, were not used. Therefore, the present findings reflect subjective sleep quality rather than objectively measured sleep parameters. Third, specific sleep disorders, including REM sleep behavior disorder, insomnia, excessive daytime sleepiness, and obstructive sleep apnea, were not examined individually. Finally, detailed information regarding antiparkinsonian medication regimens (e.g., medication type, dosage, or levodopa equivalent daily dose) was not collected. Therefore, the potential influence of pharmacological treatment on sleep quality and cognitive performance could not be evaluated. In addition, no a priori sample size calculation was performed, which may limit the statistical power and generalizability of the findings. Future studies should include detailed medication data and perform an a priori sample size calculation to better account for these methodological considerations.

## 5. Conclusions

In conclusion, Parkinson’s patients with poor sleep quality have been shown to have higher disease severity and lower global cognitive performance, executive functions, and visuospatial performance. Remarkably, these differences were also observed in early-to-middle-stage patients with PD who were largely cognitively preserved. This finding suggests that cognitive differences based on sleep quality are not limited to advanced-stage patients. Our findings indicate that sleep quality is not merely a common non-motor symptom in PD but an important feature that should be assessed in conjunction with clinical and cognitive status. The ability of this simple classification based on subjective sleep quality to reveal clinical and cognitive differences, and the ease of use of the PSQI tool for differentiating individuals with varying levels of cognitive performance, suggests that sleep assessment can be considered as part of routine clinical monitoring and holistic Parkinson’s care. It is expected that future studies incorporating objective sleep assessments and longitudinal follow-up designs will better elucidate the direction and underlying mechanisms of the relationship between sleep disturbances and cognitive changes.

## Figures and Tables

**Table 1:** Demographic characteristics of patients with Parkinson’s disease according to sleep quality.

		Good Sleep Quality		Poor Sleep Quality		*p*	Effect Size
		$\bar{X}$ ± SD	$\tilde{X}$ (Min–Max)	$\bar{X}$ ± SD	$\tilde{X}$ (Min–Max)		
Age (year)		62.31 ± 6.24	64.5 (45–70)	63.78 ± 5.71	66 (46–70)	0.217 ^a^	0.15
BMI (kg/m^2^)		25.81 ± 2.41	26.59 (20.62–29.30)	26.21 ± 2.06	26.66 (22.20–29.55)	0.462 ^b^	0.18
		***n***	**%**	***n***	**%**	***p*** **^c^**	
Gender	Female	14	43.8	15	40.5	0.788	
	Male	18	56.3	22	59.5		
Level of Education	Illiterate	3	9.4	3	8.1	0.348	
	Primary Education	9	28.1	14	37.8		
	High School	9	28.1	14	37.8		
	High Education	11	34.4	6	16.2		

($\bar{X}$) ± SD: Mean ± Standard Deviation, $\tilde{X}$ (min–max): Median (minimum–maximum), *n*: Number of Patients, BMI: Body Mass Index, ^a^: Mann–Whitney U Test (effect size reported as r), ^b^: Independent samples *t*-test (effect size reported as Cohen’s d), ^c^: Pearson chi-square test or Fisher’s exact test, as appropriate.

**Table 2:** Clinical characteristics according to sleep quality in patients with Parkinson’s disease.

		Good Sleep Quality		Poor Sleep Quality		*p*	Effect Size
		$\bar{X}$ ± SD	$\tilde{X}$ (Min–Max)	$\bar{X}$ ± SD	$\tilde{X}$ (Min–Max)		
UPDRS		31.16 ± 7.44	31.5 (18–45)	37.19 ± 9.18	39 (16–55)	0.004 ^b,^*	0.72
PSQI		3.50 ± 0.71	4 (2–5)	10.16 ± 2.75	10 (6–15)	<0.001 ^a,^*	0.87
Disease Duration (year)		5.28 ± 3.24	5.5 (1–14)	7.29 ± 4.81	7 (1–20)	0.043 ^b,^*	0.48
		***n***	**%**	***n***	**%**	***p*** **^c^**	
Dominant Extremity	Right	29	90.6	35	94.6	0.657	
	Left	3	9.4	2	5.4		
Disease Onset	Right	13	40.6	23	62.2	0.074	
	Left	19	59.4	14	37.8		
mH&Y	Stage 1.5	7	21.9	2	5.4	<0.001 *	
	Stage 2	13	40.6	5	13.5		
	Stage 2.5	11	34.4	15	40.5		
	Stage 3	1	3.1	15	40.5		

($\bar{X}$) ± SD: Mean ± Standard Deviation, $\tilde{X}$ (min–max): Median (minimum–maximum), *n*: Number of Patients, PSQI: Pittsburgh Sleep Quality Index, UPDRS: Unified Parkinson’s Disease Rating Scale, mH&Y: Modified Hoehn and Yahr Scale, ^a^: Mann–Whitney U Test (effect size reported as r), ^b^: Independent samples *t*-test (effect size reported as Cohen’s d), ^c^: Pearson chi-square test or Fisher’s exact test, as appropriate, *: *p* < 0.05.

**Table 3:** Comparison of cognitive performance according to sleep quality in patients with Parkinson’s disease.

	Good Sleep Quality		Poor Sleep Quality		*p*	Effect Size
	$\bar{X}$ ± SD	$\tilde{X}$ (Min–Max)	$\bar{X}$ ± SD	$\tilde{X}$ (Min–Max)		
MoCA	25.28 ± 1.83	25 (22–29)	24.40 ± 1.62	25 (21–28)	0.039 ^b,^*	0.51
Stroop-1 Time (sec)	11.35 ± 2.26	11.09 (7.82–18.72)	12.85 ± 3.54	12.33 (7.31–23.62)	0.039 ^b,^*	0.49
Stroop-2 Time (sec)	14.36 ± 3.07	13.76 (9.17–24.87)	16.13 ± 3.90	15.74 (8.03–26.76)	0.042 ^b,^*	0.50
Stroop-3 Time (sec)	14.96 ± 3.51	14.86 (10.11–22.54)	17.08 ± 4.36	16.21 (10.30–31.43)	0.027 ^a,^*	0.27
Stroop-4 Time (sec)	20.08 ± 3.16	18.98 (15.67–27.86)	24.19 ± 8.70	21.34 (13.21–55.34)	0.032 ^a,^*	0.26
Stroop-5 Time (sec)	28.39 ± 6.47	26.01 (20.13–46.30)	34.51 ± 12.26	31.52 (18.42–75.87)	0.011 ^a,^*	0.31
Clock Drawing Test	6.50 ± 1.52	7 (3–9)	5.54 ± 1.78	6 (1–8)	0.019 ^a,^*	0.28

($\bar{X}$) ± SD: Mean ± Standard Deviation, $\tilde{X}$ (min–max): Median (minimum–maximum), MoCA: Montreal Cognitive Assessment. ^a^: Mann–Whitney U Test (effect size reported as r), ^b^: Independent samples *t*-test (effect size reported as Cohen’s d), *: *p* < 0.05.

**Table 4:** Adjusted comparisons of cognitive performance according to sleep quality after controlling for age, disease duration, and Modified Hoehn and Yahr Scale.

	Good Sleep Quality		Poor Sleep Quality		*F*	*p* ^d^	$\eta_{p}^{2}$
	Adjusted Mean (SE)	95% CI for the Adjusted Mean	Adjusted Mean (SE)	95% CI for the Adjusted Mean			
MoCA	25.399 (0.331)	24.736–26.061	24.304 (0.305)	23.695–24.913	5.126	0.027 *	0.074
Stroop-1 Time (s)	11.173 (0.591)	9.993–12.353	13.009 (0.543)	11.923–14.094	4.536	0.037 *	0.066
Stroop-2 Time (s)	13.844 (0.667)	12.510–15.177	16.588 (0.614)	15.362–17.814	7.944	0.006 *	0.110
Stroop-3 Time (s)	14.621 (0.755)	13.113–16.130	17.377 (0.694)	15.989–18.764	6.257	0.015 *	0.089
Stroop-4 Time (s)	19.576 (1.304)	16.971–22.181	24.644 (1.199)	22.249–27.040	7.101	0.010 *	0.100
Stroop-5 Time (s)	27.528 (1.950)	23.633–31.423	35.264 (1.793)	31.682–38.846	7.399	0.008 *	0.104
Clock Drawing Test	6.594 (0.321)	5.953–7.235	5.459 (0.295)	4.870–6.049	5.875	0.018 *	0.084

SE: Standard Error, CI: Confidence Interval, *η*^2^*p*: Partial eta squared, MoCA: Montreal Cognitive Assessment. ^d^: *p*-values were obtained from analysis of covariance (ANCOVA) adjusted for age, disease duration, and Modified Hoehn and Yahr Scale (mH&Y), *: *p* < 0.05.

## Data Availability

The datasets generated and analyzed during the current study are not publicly available for ethical reasons but are available from the corresponding author upon reasonable request.

## Supplementary Materials

The following supporting information can be downloaded at https://www.mdpi.com/article/10.3390/healthcare14162455/s1. Table S1: Regression coefficients from the ANCOVA models evaluating cognitive performance after adjustment for age, disease duration, and Modified Hoehn and Yahr stage; Table S2: Multicollinearity diagnostics; Table S3: Distributional characteristics of the raw outcome variables by sleep-quality group; Table S4: Residual diagnostics for the ANCOVA models; Table S5: Sensitivity analyses using log-transformed Stroop scores.

## Abbreviations

The following abbreviations are used in this manuscript:

BMI	Body Mass Index
mH&Y	Modified Hoehn and Yahr Scale
MoCA	Montreal Cognitive Assessment
PD	Parkinson’s Disease
PSQI	Pittsburgh Sleep Quality Index
SD	Standard Deviation
STROBE	Strengthening the Reporting of Observational Studies in Epidemiology
TBAG	Turkish Psychology Working Group
UPDRS	Unified Parkinson’s Disease Rating Scale
