# Supplementary material for: The Relevance of Sleep Quality for Clinical Care in Parkinson’s Disease: Clinical and Cognitive Differences Between Good and Poor Sleepers: A Cross-Sectional Study
Source: Healthcare (Basel). 2026 Aug 9;14(16):2455. doi: 10.3390/healthcare14162455 (PMC13512637; doi:10.3390/healthcare14162455)
Supplement: Supplementary file 1 [file healthcare-14-02455-s001.zip › healthcare-4415839-supplementary.pdf]

Supplementary Material

Supplementary Table S1. Regression coefficients from the ANCOVA models evaluating cognitive performance after adjustment for age, disease duration, and Modified Hoehn and Yahr stage.

Outcome	Predictor	B	SE	t	p	95% CI
MoCA	Group	1.095	0.483	2.264	0.027	0.129 to 2.060
	Age	0.039	0.035	1.116	0.269	-0.031 to 0.109
	Disease duration	0.080	0.052	1.544	0.128	-0.023 to 0.183
	mH&Y	0.000	0.509	0.000	1.000	-1.018 to 1.018
Stroop-1	Group	-1.835	0.862	-2.130	0.037	-3.557 to -0.114
	Age	-0.067	0.063	-1.063	0.292	-0.192 to 0.059
	Disease duration	-0.007	0.092	-0.081	0.936	-0.192 to 0.177
	mH&Y	-0.470	0.908	-0.518	0.607	-2.284 to 1.344
Stroop-2	Group	-2.744	0.974	-2.819	0.006	-4.689 to -0.799
	Age	-0.112	0.071	-1.580	0.119	-0.253 to 0.030
	Disease duration	-0.138	0.104	-1.323	0.190	-0.346 to 0.070
	mH&Y	-1.089	1.026	-1.062	0.292	-3.139 to 0.960
Stroop-3	Group	-2.755	1.102	-2.501	0.015	-4.956 to -0.555
	Age	-0.179	0.080	-2.239	0.029	-0.339 to -0.019
	Disease duration	0.052	0.118	0.443	0.659	-0.183 to 0.288
	mH&Y	-0.980	1.161	-0.844	0.402	-3.298 to 1.339
Stroop-4	Group	-5.069	1.902	-2.665	0.010	-8.869 to -1.269
	Age	-0.157	0.138	-1.137	0.260	-0.434 to 0.119
	Disease duration	0.146	0.203	0.716	0.476	-0.261 to 0.552
	mH&Y	-2.095	2.004	-1.045	0.300	-6.099 to 1.909
Stroop-5	Group	-7.736	2.844	-2.720	0.008	-13.418 to -2.054
	Age	-0.189	0.207	-0.915	0.364	-0.602 to 0.224
	Disease duration	0.059	0.304	0.193	0.847	-0.549 to 0.666
	mH&Y	-2.994	2.997	-0.999	0.321	-8.981 to 2.992
Clock Drawing Test	Group	1.134	0.468	2.424	0.018	0.199 to 2.069
	Age	0.048	0.034	1.418	0.161	-0.020 to 0.116
	Disease duration	0.068	0.050	1.358	0.179	-0.032 to 0.168
	mH&Y	-0.068	0.493	-0.138	0.891	-1.053 to 0.917

Supplementary Table S2. Multicollinearity diagnostics

Predictor	Tolerance	VIF
Sleep-quality group	0.733	1.363
Age	0.983	1.018
Disease duration	0.895	1.118
Modified Hoehn & Yahr	0.706	1.416

Note. All VIF values were below 2.0 and all tolerance values exceeded 0.70, indicating no evidence of problematic multicollinearity.

Supplementary Table S3. Distributional characteristics of the raw outcome variables by sleep-quality group

Outcome	Group	Shapiro–Wilk (W)	p	Skewness	Kurtosis
MoCA	Good sleep	0.941	0.078	0.390	0.045
	Poor sleep	0.957	0.159	0.035	-0.557
Stroop-1 Time (s)	Good sleep	0.924	0.028	1.156	2.170
	Poor sleep	0.922	0.013	1.106	1.471
Stroop-2 Time (s)	Good sleep	0.919	0.019	1.235	3.029
	Poor sleep	0.983	0.819	0.442	0.328
Stroop-3 Time (s)	Good sleep	0.923	0.025	0.659	-0.411
	Poor sleep	0.925	0.016	1.130	2.052
Stroop-4 Time (s)	Good sleep	0.918	0.019	0.817	-0.219
	Poor sleep	0.835	<0.001	1.853	4.446
Stroop-5 Time (s)	Good sleep	0.875	0.001	1.277	1.526
	Poor sleep	0.824	<0.001	1.784	3.391
Clock Drawing Test	Good sleep	0.896	0.005	-0.700	-0.396
	Poor sleep	0.925	0.016	-0.639	-0.118

Note. Distributional characteristics of the raw outcome variables were evaluated using Shapiro–Wilk tests together with skewness and kurtosis statistics, histograms, and Q–Q plots. The selection of the independent-samples t test or Mann–Whitney U test for the unadjusted comparisons in Table 3 was based on the overall assessment of these distributional characteristics rather than on the Shapiro–Wilk test alone.

Supplementary Table S4. Residual diagnostics for the ANCOVA models

Table. Residual skewness and kurtosis values of the standardized residuals for each ANCOVA model. Approximate normality was additionally evaluated by visual inspection of histograms and normal Q–Q plots.

Outcome	Residual skewness	Residual kurtosis	Visual inspection
MoCA	0.336	0.130	Acceptable
Stroop-1	1.041	2.252	Acceptable
Stroop-2	0.240	0.469	Acceptable
Stroop-3	0.503	0.401	Acceptable
Stroop-4	1.806	5.933	Slight right-skew caused by a small number of high residuals
Stroop-5	1.713	3.577	Slight right-skew caused by a small number of high residuals
Clock Drawing Test	-0.619	-0.158	Acceptable

Note. Approximate normality of the standardized residuals was evaluated using residual skewness and kurtosis statistics together with visual inspection of histograms and normal Q–Q plots.

Supplementary Table S5. Sensitivity analyses using log-transformed Stroop scores

Table note. Sensitivity analyses were performed because Stroop-4 and Stroop-5 exhibited the greatest departure from normality. ANCOVA models were repeated using natural log-transformed scores while adjusting for age, disease duration, and Modified Hoehn and Yahr stage. The statistical significance of the sleep-quality group effect was unchanged, indicating that the primary findings were robust to the distributional characteristics of these variables.

Dependent variable	ANCOVA p (Table 4)	Log-transformed ANCOVA F	Log-transformed ANCOVA p	Interpretation
Stroop-4	0.010	5.697	0.020	Group effect remained statistically significant
Stroop-5	0.008	7.254	0.009	Group effect remained statistically significant
